# ID 36 - Mapeamento de Iniciativas Educacionais para Treinamento em Síntese de Evidências, Avaliação Econômica e Desenvolvimento de Diretrizes Clínicas no Brasil

**DOI:** 10.5327/2237-9622.2025.v34s1.36

**Published:** 2025-11-25

**Authors:** Bruna Marmett, Ana Paula Blankenheim, Bárbara Cristiane da Silva, Rodrigo Pereira de Almeida, Roseana Boek Carvalho, Marina Petrasi Guahnon, Muriel Primon de Barros, Cinara Stein, Gilson Pires Dorneles, Suena Medeiros Parahiba, Maicon Falavigna

**Keywords:** iniciativas educacionais, síntese de evidência, tomada de decisão

## Abstract

**Introdução:**

A síntese de evidências e a avaliação econômica fornecem informações para a tomada de decisões que apoiam o avanço de um sistema de saúde equitativo. Em países de baixa e média renda há a falta de profissionais especializados no tema. Fato que eleva a necessidade de estratégias educacionais para aumentar a disseminação de conhecimento sobre síntese de evidências e desenvolvimento de diretrizes de prática clínica (DPC). O objetivo é mapear os cursos on-line disponíveis para os tópicos de síntese de evidências, avaliação econômica e diretrizes clínicas oferecidos no Brasil.

**Método:**

Foi realizada uma revisão de escopo de fevereiro a maio de 2024, buscando iniciativas educacionais nos sites das agências brasileiras de ATS, nas bases de dados PubMed/MEDLINE, LILACS e no Google. Foram incluídos cursos on-line desenvolvidos ou ofertados no Brasil, em português, sem limite de data de oferta. Dois revisores independentes incluíram cursos on-line que correspondiam à população, conceito e contexto (PCC). A extração de dados incluiu: público-alvo, modalidade, duração, tipo de acesso, região, taxa, ano de lançamento, carga horária, objetivos, conteúdos programáticos, métodos de avaliação e disponibilidade de materiais de leitura complementares. Registro do protocolo: 10.17605/OSF.IO/3E7DQ.

**Resultados:**

Quarenta e seis cursos atenderam aos critérios de inclusão e foram categorizados nos principais temas de síntese de evidências (n=28), avaliação econômica (n=10) e diretrizes de prática clínica (n=8). O público-alvo mais frequentemente visado compreendia profissionais públicos e privados envolvidos em ATS (64,04%). A maioria dos cursos tinha carga horária de até 20 horas (58,7%), era autoinstrucional (63,04%), assíncrono (47,82%) e oferecido gratuitamente (89,96%). As agências patrocinadoras desses cursos estavam concentradas em São Paulo, Rio Grande do Sul, Distrito Federal e Goiás.

**Conclusão:**

Há um número significativo de iniciativas educacionais focadas em ATS, e mais cursos em tópicos como avaliação econômica são necessários para aprimorar as habilidades profissionais e fortalecer um de tomada de decisão mais robusto. Apesar disso, mitigar disparidades regionais e expandir a oferta de cursos são passos essenciais para promover uma força de trabalho em saúde equitativa e bem informada em todo o País.

